# Relevance of the correlation between tomography findings and laboratory test results in the accuracy of the diagnosis of pulmonary tuberculosis

**DOI:** 10.1590/0100-3984.2023.0079-en

**Published:** 2024-05-07

**Authors:** Daniel Lopes da Cunha, Maria Lucia Rossetti, Evaldo Teixeira Nunes, Eduardo Bruno Lobato Martins, Aila de Menezes Ferreira, Sariane Coelho Ribeiro

**Affiliations:** 1 Hospital Universitário da Universidade Federal do Piauí (UFPI), Teresina, PI, Brazil; 2 Universidade Luterana do Brasil (Ulbra), Canoas, RS, Brazil; 3 Clínica UDI 24 Horas, Teresina, PI, Brazil

**Keywords:** Tuberculosis, pulmonary, Multidetector computed tomography, Diagnostic techniques, respiratory system, Clinical laboratory techniques, Molecular diagnostic techniques, Sputum/microbiology, Tuberculose pulmonar, Tomografia computadorizada multidetectores, Técnicas de diagnóstico do sistema respiratório, Técnicas de laboratório clínico, Técnicas de diagnóstico molecular, Escarro/microbiologia

## Abstract

**Objective:**

To evaluate the correlation between multidetector computed tomography (MDCT) findings and laboratory test results in patients with pulmonary tuberculosis (PTB).

**Materials and Methods:**

A total of 57 patients were evaluated. Patients with suspected PTB were divided into groups according to the final diagnosis (confirmed or excluded), and the groups were compared in terms of sociodemographic variables, clinical symptoms, tomography findings, and laboratory test results.

**Results:**

Among the patients with a confirmed diagnosis of PTB, small pulmonary nodules with a peribronchovascular distribution were significantly more common in the patients with a positive sputum smear microscopy result (47.4% vs. 8.3%; *p* = 0.046), as were a miliary pattern (36.8% vs. 0.0%; *p* = 0.026), septal thickening (84.2% vs. 41.7%; *p* = 0.021), and lymph node enlargement (52.6% vs. 8.3%; *p* = 0.020). Small pulmonary nodules with a centrilobular distribution were significantly more common among the culture-positive patients (75.0% vs. 35.7%; *p* = 0.045), as was a tree-in-bud pattern (91.7% vs. 42.9%; *p* = 0.014). A tree-in-bud pattern, one of the main tomography findings characteristic of PTB, had a sensitivity, specificity, positive predictive value, and negative predictive value of 71.0%, 73.1%, 75.9%, and 67.9%, respectively.

**Conclusion:**

MDCT presented reliable predictive values for the main tomography findings in the diagnosis of PTB, being a safe tool for the diagnosis of PTB in patients with clinical suspicion of the disease. It also appears to be a suitable tool for the selection of patients who are candidates for more complex, invasive examinations from among those with high clinical suspicion of PTB and a negative sputum smear microscopy result.

## Abstract (pt)

**Objetivo:**

Avaliar a correlação entre os achados na tomografia computadorizada multidetectores (TCMD) comparativamente aos resultados laboratoriais em pacientes com tuberculose pulmonar (TBP).

**Materiais e Métodos:**

Amostra de 57 pacientes foi avaliada. Pacientes com suspeita clínica de TBP foram divididos de acordo com a positividade do diagnóstico, e as variáveis sociodemográficas, sintomas clínicos e achados tomográficos e laboratoriais foram comparados.

**Resultados:**

Nos pacientes com TBP e baciloscopia positiva, foram verificadas frequências significativas para pequenos nódulos pulmonares com distribuição peribroncovascular (47,4% vs. 8,3%; *p* = 0,046) e miliar (36,8% vs. 0,0%; *p* = 0,026), espessamento septal (84,2% vs. 41,7%; *p* = 0,021) e linfonodomegalias (52,6% vs. 8,3%; *p* = 0,020). Em relação à cultura, os pequenos nódulos pulmonares com distribuição centrolobular (75,0% vs. 35,7%; *p* = 0,045) e opacidades em árvore em brotamento (91,7% vs. 42,9%; *p* = 0,014) apresentaram frequências significativamente superiores. Medidas de sensibilidade, especificidade, valor preditivo positivo e valor preditivo negativo para árvore em brotamento, um dos principais achados tomográficos característicos da TBP, foram, respectivamente, 71.0%, 73,1%, 75,9% e 67,9%.

**Conclusão:**

A TCMD apresentou medidas preditivas confiáveis para os principais achados tomográficos no diagnóstico de TBP, sendo uma ferramenta segura para o diagnóstico da doença em pacientes com suspeita clínica. Também se mostrou adequada para selecionar os pacientes para exames mais complexos e invasivos entre os com alta suspeita clínica de TBP e baciloscopia negativa.

## INTRODUCTION

Tuberculosis is an airborne disease caused by *Mycobacterium tuberculosis*^(1)^. In 2021, approximately 10 million people worldwide contracted tuberculosis; of those, 1.3 million died, with 214,000 of those deaths occurring among HIV-infected individuals^(2)^. The greatest difficulties faced in combating tuberculosis have been delays in diagnosis and in the start of treatment^(3)^, despite the fact that the treatment is affordable and effective^(4)^.

The coronavirus pandemic of 2020 and 2021 had enormous health, social, and economic impacts, limiting the availability of and access to essential services for the diagnosis and treatment of tuberculosis^(2)^. Failure to diagnose and treat affected patients in a timely manner leads to increased morbidity and mortality, the development of secondary resistance, and continued transmission of the disease^(5)^.

Although sputum smear microscopy provides benefits in terms of cost and time, it has low sensitivity^(6)^-from 22-43% for a single smear up to 60% under optimal conditions-in comparison with sputum culture^(7)^. The GeneXpert MTB/RIF test, which is a rapid molecular test for *M. tuberculosis* and for rifampin resistance, uses polymerase chain reaction (PCR) to detect *M. tuberculosis* by the nucleic acid amplification method, providing results in approximately two hours^(6)^. In cases of smear-positive and smear-negative pulmonary tuberculosis (PTB), the GeneXpert MTB/RIF test has a sensitivity of 98.2% and 72.5%, respectively^(7)^. Despite being the gold standard for detecting and diagnosing PTB, sputum culture does not provide a quick diagnosis, taking four to eight weeks to provide a result^(6,8)^. In comparison with chest X-ray, multidetector computed tomography (MDCT) of the chest is more sensitive and better facilitates the differential diagnosis of lung parenchymal lesions, as well as allowing a more accurate assessment of disease activity and complications^(9)^. Therefore, MDCT constitutes a particularly valuable method for use in smear-negative patients with suspected PTB^(10)^.

The role of MDCT in managing treatment and investigating complications in patients with PTB is well recognized. However, there have been few studies of the correlation between the main tomography findings described in patients with PTB and the results of diagnostic laboratory tests. There have also been few studies describing the predictive values of the main tomography findings in patients with PTB and their importance in the management of the disease, especially in areas where it is highly prevalent and there are few public resources to combat it.

This primary objective of this study was to evaluate the correlation between MDCT findings and laboratory test results in patients with suspected PTB and to demonstrate that MDCT presents reliable predictive measures for the diagnosis of the disease.

## MATERIALS AND METHODS

This was a cross-sectional analytical study, designed to evaluate the relationship between MDCT findings and laboratory test results in patients with PTB. The study was carried out between September 2018 and March 2020 in the Imaging Department of the Hospital Universitário da Universidade Federal do Piauí (HU-UFPI), in the city of Teresina, Brazil.

We evaluated 67 patients with suspected PTB who presented one or more of the following symptoms: cough (dry or productive); hemoptysis; chest pain; and constitutional symptoms, such as weight loss, fever (> 38.5°C), night sweats, and dyspnea. Patients who did not undergo chest MDCT or laboratory tests for diagnosis were excluded, as were those who were currently undergoing or had previously undergone treatment for PTB. The final sample comprised 57 patients. The study was approved by the HU-UFPI Research Ethics Committee (Reference no. 2,878,866-2018), and all participating patients gave written informed consent.

**Figure 1:**
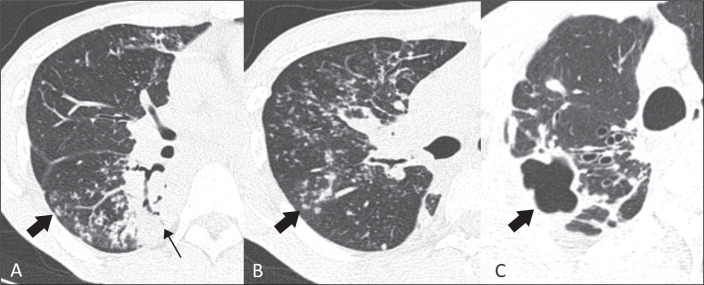
Main MDCT findings related to PTB. A: Consolidation (thin arrow), characterized by increased attenuation of the lung parenchyma, which prevents visualization of the vessels and the external contours of the bronchial walls, although air bronchograms can be seen. Note the tree-in-bud pattern (thick arrow), characterized by centrilobular branching opacities, with small nodulations at the ends, resembling the sprouting of trees and indicative of dilated bronchioles filled with pathological material. B: Centrilobular nodular pattern (arrow). Distribution of small nodules occupying the central portion of the secondary pulmonary lobule, typically related to bronchiolar disease. (If this is accompanied by a tree-in-bud pattern, infectious causes should be considered.) C: Cavitation (arrow), characterized by a gas-filled space, with or without an air-fluid level, within a pulmonary consolidation, with irregular contours and a thickness of more than 1 mm.

Chest MDCT was performed in a 16-slice scanner (Somatom Emotion 16; Siemens Healthineers, Erlangen, Germany). The MDCT findings were analyzed by a radiologist with 18 years of experience, working independently, who was blinded to the clinical symptoms and laboratory test results. The scans were evaluated for the presence or absence and extent of the following findings (Figure 1): small pulmonary nodules (< 10 mm); a tree-in-bud pattern; large pulmonary nodules (10-30 mm); pulmonary mass (> 30 mm); ground-glass opacity; consolidation; cavitation; bronchial wall thickening; septal thickening; fibrotic opacities distorting the pulmonary architecture; pleural effusion; mediastinal lymph node enlargement (short axis > 10 mm); and mediastinal lymph node enlargement with central necrosis.

Sputum smear microscopy and molecular tests, including the GeneXpert MTB/RIF test and culture on solid medium, were carried out following the recommendations of the Brazilian National Ministry of Health^(11)^. Patients were considered to have a confirmed diagnosis of PTB if they had two positive sputum smear microscopy results; a positive sputum smear microscopy result and a positive culture; a positive sputum smear microscopy result and radiological imaging findings suggestive of PTB; or a negative sputum smear microscopy result and a positive molecular test or positive culture. Patients with suspected PTB were divided into two groups-those with and without a confirmed diagnosis-and the two groups were compared in terms of sociodemographic variables, clinical symptoms, and MDCT findings. Patients with confirmed PTB were divided into groups according to the result (positivity or negativity) on each laboratory test (sputum smear microscopy, molecular test, and culture), and the relationships between the frequency of MDCT findings and positivity on those tests were analyzed. The sensitivity, specificity, positive predictive value (PPV), and negative predictive value (NPV) of the main MDCT findings were also calculated.

In the inferential analysis, we correlated each MDCT finding with each laboratory test result, using Pearson’s chi-square test or Fisher’s exact test for frequency trends. Values of *p* < 0.05 were considered statically significant. In the analysis of the sensitivity, specificity, PPV, and NPV of the MDCT findings, we used the Analyse-it program.

## RESULTS

In our sample of patients with suspected PTB, the mean age was 50.7 ± 18.2 years (range, 21.3-89.0 years). Of the 57 patients evaluated, 35 (61.4%) were men. The diagnosis of PTB was confirmed in 31 (54.4%) of the patients, 22 (71.0%) of whom were male. However, as shown in Table 1, there was no significant association between gender and the occurrence of PTB (*p* = 0.105).

**Table 1:** Sociodemographic characteristics and clinical symptoms of patients with suspected PTB.

Variable	Diagnosis of PTB		Total (N = 57)	*P*
	Confirmed (n = 31)	Excluded (n = 26)		
Age (years), mean ± SD	53.7 ± 19.6	47.1 ± 15.9	50.7 ± 18.2	0.169^*^
Sex, n (%)				0.105^‡^
Male	22 (71.0%)	13 (50.0%)	35 (61.4%)	
Female	9 (29,0%)	13 (50.0%)	22 (38.6%)	
Symptoms, n (%)^*^				
Cough	22 (71.0%)	20 (76.9%)	42 (73.7%)	0.611^*^
Expectoration	9 (29.0%)	7 (26.9%)	16 (28.1%)	0.860^*^
Hemoptysis	6 (19.4%)	5 (19.2%)	11 (19.3%)	0.991^*^
Chest pain	6 (19.4%)	15 (57.7%)	21 (36.8%)	0.003^*^
Fever (> 38.5°C)	19 (61.3%)	10 (38.5%)	29 (50.9%)	0.086^*^
Night sweats	1 (3.2%)	5 (19.2%)	6 (10.5%)	0.083^§^
Weight loss	15 (48.4%)	16 (61.5%)	31 (54.4%)	0.321^*^
Dyspnea	11 (35.5%)	9 (34.6%)	20 (35.1%)	0.945^*^

* Frequencies only for the “yes” category.

† Student’s t-test.

‡ Pearson’s chi-square test.

§ Fisher’s exact test.

The frequency of symptoms was higher among the patients with a confirmed diagnosis of PTB than among those without: expectoration (29.0% vs. 26.9%), hemoptysis (19.4% vs. 19.2%), fever (61.3% vs. 38.5%) and dyspnea (35.5% vs. 34.6%). However, the only significant difference was related to the symptom of chest pain, which was more common among those in whom a diagnosis of PTB was ruled out (57.7% vs. 19.4%; *p* = 0.003).

Of the 31 patients with a confirmed diagnosis of PTB, 19 (61.3%) had a positive sputum smear microscopy result, 28 (90.3%) had a positive GeneXpert MTB/RIF test result, and 14 (46.2%) had a positive culture for *M. tuberculosis* (Table 2).

**Table 2:** Laboratory test results and MDCT findings in patients with suspected PTB.

Characteristic	Diagnosis of TBP		Total (N = 57) n (%)	*P*
	Confirmed (n = 31) n (%)	Excluded (n = 26) n (%)		
Positive sputum smear microscopy result	19 (61.3%)	0 (0.0%)	19 (33.3%)	<0.001^*^
Positive GeneXpert MTB/RIF result	28 (90.3%)	0 (0.0%)^b^	28 (50.0%)^c^	<0.001^‡^
Positive culture	12 (46.2%)^a^	0 (0.0%)^b^	12 (23.5%)^d^	<0.001^*^
MDCT findings^*^				
Small pulmonary nodules (< 10 mm)	18 (58.1%)	6 (23.1%)	24 (42.1%)	0.008^*^
Centrilobular	18 (58.1%)	6 (23.1%)	24 (42.1%)	0.008^*^
Perilymphatic	17 (54.8%)	5 (19.2%)	22 (38.6%)	0.006^*^
Peribronchovascular	10 (32.3%)	2 (7.7%)	12 (21.1%)	0.023^*^
Septal	11 (35.5%)	3 (11.5%)	14 (24.6%)	0.036^*^
Subpleural	14 (45.2%)	5 (19.2%)	19 (33.3%)	0.039^*^
Random (miliary) distribution	7 (22.6%)	1 (3.8%)	8 (14.0%)	0.059^§^
Tree-in-bud pattern	22 (71%)	7 (26.9%)	29 (50.9%)	0.001^*^
Large pulmonary nodules (10-30 mm)	3 (9.7%)	2 (7.7%)	5 (8.8%)	0.999^§^
Lung mass (> 30 mm)	2 (6.5%)	0 (0%)	2 (3.5%)	0.495^§^
Ground-glass opacity	14 (45.2%)	9 (34.6%)	23 (40.4%)	0.419^*^
Consolidation	19 (61.3%)	7 (26.9%)	26 (45.6%)	0.009^*^
Cavitation	14 (45.2%)	4 (15.4%)	18 (31.6%)	0.016^*^
Bronchial wall thickening	19 (61.3%)	4 (15.4%)	23 (40.4%)	<0.001^*^
Septal thickening	21 (67.7%)	10 (38.5%)	31 (54.4%)	0.027^*^
Fibrotic opacities/distortion of lung architecture	23 (74.2%)	12 (46.2%)	35 (61.4%)	0.030^*^
Pleural effusion	15 (48.4%)	4 (15.4%)	19 (33.3%)	0.008^*^
Lymph node enlargement (short axis > 1 cm)	11 (35.5%)	6 (23.1%)	17 (29.8%)	0.308^*^
Lymph node enlargement with central necrosis	2 (6.5%)	0 (0%)	2 (3,5%)	0.495^§^

* Frequencies only for the “yes” category.

† Pearson’s chi-square test.

‡ Fisher’s exact test.

§ Student’s t-test.

a Data available for only 26 patients.

b Data available for only 25 patients.

c Data available for only 56 patients.

d Data available for only 51 patients.

The following findings were significantly more common among the patients with a confirmed diagnosis of PTB than among those without (Table 2): small pulmonary nodules (58.1% vs. 23.1%; *p* = 0.008), consolidation (61.3% vs. 26.9%; *p* = 0.009), cavitation (45.2% vs. 15.4%; *p* = 0.016) and a tree-in-bud pattern (71.0% vs. 26.9%; *p* = 0.001). Among the patients with small pulmonary nodules, centrilobular nodules were more common in those with a confirmed diagnosis of PTB (58.1% vs. 23.1%; *p* = 0.008).

Table 3 compares the patients in relation to the MDCT findings, by the results of the laboratory tests. The following findings were significantly more common among the patients who were smear-positive than among those who were smear-negative: small pulmonary nodules with a peribronchovascular distribution (47.4% vs. 8.3%; *p* = 0.046); small pulmonary nodules with a miliary pattern (36.8% vs. 0.0%; *p* = 0.026); septal thickening (84.2% vs. 41.7%; *p* = 0.021); and lymph node enlargement (52.6% vs. 8.3%; *p* = 0.020). None of the MDCT findings were significantly associated with positivity on the GeneXpert MTB/RIF test (*p* > 0.05 for all). Small pulmonary nodules with a centrilobular distribution were significantly more common among the patients who were culture-positive than among those who were culture-negative (75.0% vs. 35.7%; *p* = 0.045), as was a tree-in-bud pattern (91.7% vs. 42.9%; *p* = 0.014).

**Table 3:** Comparison of the frequencies of MDCT findings in patients diagnosed with PTB, by the results of laboratory tests

MDCT findingss^*^	Sputum smear microscopy (n = 31)			GeneXpert MTB/RIF^*^ (n = 31)			Culture (n = 26)		
	Positive (n = 19) n (%)	Negative (n = 12) n (%)	*P*	Positive (n = 28) n (%)	Negative (n = 3) n (%)	*P*	Positive (n = 12) n (%)	Negative (n = 14) n (%)	*P*
Small pulmonary nodules	13 (68.4)	5 (41.7)	0.141^‡^	18 (64.3)	0 (0.0)	0,064^§^	7 (58.3)	6 (42.9)	0.431^*^
Centrilobular	13 (68.4)	5 (41.7)	0.130^§^	18 (64.3)	1 (33.3)	0,543^§^	9 (75.0)	5 (35.7)	0.045^*^
Perilymphatic	13 (68.4)	4 (33.3)	0.056^*^	17 (60.7)	0 (0.0)	0,081^§^	7 (58.3)	6 (42.9)	0.431^*^
Peribronchovascular	9 (47.4)	1 (8.3)	0.046^§^	10 (35.7)	0 (0.0)	0,533^§^	3 (25.0)	3 (21.4)	1.000^§^
Septal	9 (47.4)	2 (16.7)	0.128^§^	11 (39.3)	0 (0.0)	0,535^§^	5 (41.7)	4 (28.6)	0.683^§^
Subpleural	11 (57.9)	3 (25.0)	0.073^*^	14 (50.0)	0 (0.0)	0,232^§^	7 (58.3)	4 (28.6)	0.126^*^
Random (miliary) distribution	7 (36.8)	0 (0.0)	0.026^§^	7 (25.0)	0 (0.0)	1,000^§^	4 (33.3)	2 (14.3)	0.365^§^
Tree-in-bud pattern	16 (84.2)	6 (50.0)	0.056^§^	21 (75.0)	1 (33.3)	0,195^§^	11 (91.7)	6 (42.9)	0.014^§^
Large pulmonary nodules (10-30 mm)	3 (15.8)	0 (0.0)	0.265^§^	3 (10.7)	0 (0.0)	1,000^§^	2 (16.7)	1(7-1)	0.580^§^
Lung mass (> 30 mm)	0 (0.0)	2 (16.7)	0.142^§^	2 (7.1)	0 (0.0)	1,000^§^	0 (0.0)	2 (14.3)	0.483^§^
Ground-glass opacity	10 (52.6)	4 (33.3)	0.293^*^	14 (50.0)	0 (0.0)	0,232^§^	7 (58.3)	6 (42.9)	0.431^*^
Consolidation	13 (68.4)	6 (50.0)	0.452^§^	18 (64.3)	1 (33.3)	0,543^§^	9 (75.0)	7 (50.0)	0.248^§^
Cavitation	11 (57.9)	3 (25.0)	0.073^*^	12 (42.9)	2 (66.7)	0,576^§^	8 (66.7)	4 (28.6)	0.052^*^
Bronchial wall thickening	13 (68.4)	6 (50.0)	0.452^§^	18 (64.3)	1 (33.3)	0,543^§^	9 (75.0)	7 (50.0)	0.248^§^
Septal thickening	16 (84.2)	5 (41.7)	0.021^§^	19 (67.9)	2 (66.7)	1,000^§^	10 (83.3)	9 (64.3)	0.391^§^
Fibrotic opacities	14 (73.7)	9 (75.0)	1.000^§^	20 (71.4)	3 (100.0)	0,550^§^	10 (83.3)	11 (78.6)	1.000^§^
Pleural effusion	10 (52.6)	5 (41.7)	0.552^*^	14 (50.0)	1 (33.3)	1,000^§^	6 (50.0)	6 (42.9)	0.716^*^
Lymph node enlargement	10 (52.6)	1 (8.3)	0.020^§^	10 (35.7)	1 (33.3)	1,000^§^	5 (41.7)	5 (35.7)	1.000^§^
Lymph node enlargement with central necrosis	2 (10.5)	0 (0.0)	0.510^§^	2 (7.1)	0 (0.0)	1,000^§^	0 (0.0)	2 (14.3)	0.483^§^

* Frequencies only for the “yes” category.

† Molecular test.

‡ Pearson’s chi-square test.

§ Fisher’s exact test.

In the predictive analysis, individual MDCT findings were found to have the following sensitivity, specificity, PPV, and NPV, respectively, for a diagnosis of PTB (Table 4): a tree-in-bud pattern (71.0%, 73.1%, 75.9%, and 67.9%); small centrilobular pulmonary nodules (61.3%, 80.8%, 79.2%, and 63.6%); cavitation (45.2%, 84.6%, 77.8%, and 56.4%); and consolidation (61.3%, 73.1%, 73.1%, and 61.5%).

**Table 4:** Predictive analyses of MDCT findings in patients with suspected PTB.

MDCT findings	Sen.	Spe.	VPP	VPN
Small pulmonary nodules	58.1	76.9	75.0	60.6
Centrilobular	61.3	80.8	79.2	63.6
Perilymphatic	54.8	80.8	77.3	60.0
Peribronchovascular	32.3	92.3	83.3	53.3
Septal	35.5	88.5	78.6	53.5
Subpleural	45.2	80.8	73.7	55.3
Random distribution (miliary)	22.6	96.1	87.5	51.0
Tree-in-bud pattern	71.0	73.1	75.9	67.9
Large pulmonary nodules (10-30 mm)	9.7	92.3	60.0	46.1
Lung mass (> 30 mm)	6.4	100.0	100.0	47.3
Ground-glass opacity	45.2	65.4	60.9	50.0
Consolidation	61.3	73.1	73.1	61.3
Cavitation	45.2	84.6	77.8	56.4
Bronchial wall thickening	61.3	84.6	82.6	64.7
Septal thickening	67.7	61.5	67.7	61.5
Fibrotic opacities	74.2	53.8	65.7	63.6
Pleural effusion	48.4	84.6	78.9	57.9
Lymph node enlargement	35.5	76.9	64.7	50.0
Lymph node enlargement with central necrosis	6.4	100.0	100.0	47.3

Sen., sensitivity; Spe., specificity.

## DISCUSSION

Of the 31 patients with a confirmed diagnosis of PTB in our study, 19 (61.3%) were smear-positive, within the range of what would be expected, given that sputum smear microscopy has a sensitivity of approximately 60% under ideal conditions^(6)^. We observed a high proportion of patients with a positive PCR result (90.3%), which confirms the high sensitivity of the GeneXpert MTB/RIF test, which has been reported to be up to 98.2% in cases of smear-positive PTB and 72.5% in cases of smear-negative PTB^(7,12-14)^.

The main tomography findings described as being related to active PTB are consolidation, cavitation, small centrilobular pulmonary nodules, and a tree-in-bud pattern, the last two being consistent with endobronchial dissemination of the disease^(14)^. In the present study, small centrilobular pulmonary nodules, a tree-in-bud pattern, consolidation, and cavitation correlated significantly with a confirmed diagnosis of PTB. These findings corroborate data in the literature indicating that MDCT can be used as a means of diagnosing active PTB^(15)^.

The identification of patients with positive smear microscopy results through tomography studies would facilitate the effective isolation of these patients, who should be a high priority in PTB control policies. In the present study, we found that a positive smear microscopy result was significantly associated with the frequency of small pulmonary nodules (with peribronchovascular distribution or a miliary pattern), septal thickening, and lymph node enlargement, none which are classically described in patients with smear-positive active PTB.

Among the patients in our sample with a confirmed diagnosis of PTB who were smear-negative, we observed small centrilobular pulmonary nodules in 41.7%, a tree-in-bud pattern in 50.0%, consolidation in 50.0%, and cavitation in 25.0%, compared with 68.4%, 84.2%, and 57.9%, respectively, for those who were smear-positive, demonstrating that tomography findings typical of PTB are less pronounced when the sputum smear microscopy result is negative, which translates to a lower mycobacterial load. Because patients who are smear-negative have a lower mycobacterial load, they may not present with the clinical and radiographic findings that are typical of PTB^(14)^. Among patients with high suspicion of active PTB who are smear-negative, MDCT can facilitate the selection of candidates for additional laboratory tests or bronchoscopy, and in some cases the decision of whether to initiate antituberculosis therapy while awaiting the culture results^(16)^, especially for patients in whom there are MDCT findings that are typical of PTB, even if there are only a few such findings.

Yeh et al.^(15)^ evaluated the use of CT to predict culture-positive PTB in 4,140 adult patients with pulmonary lesions, using a set of tomography findings and their pattern of distribution in the lung parenchyma. The authors found CT to have a sensitivity, specificity, PPV, and NPV for predicting a positive culture of 98.5%, 99.7%, 92.2%, and 99.9%, respectively. Their data indicate that CT is a viable tool for identifying culture-positive PTB in an emergency setting.

Ko et al.^(17)^ evaluated the correlation between microbiology findings and radiographic activity on chest CT in patients with suspected PTB. The authors found radiographic activity (cavitation, a tree-in-bud pattern, and multiple noncalcified nodules, collectively) to have high specificity (97.1%) and a high NPV (92.7%). In the present study, we evaluated each of the main MDCT findings in isolation and found that, for the diagnosis of active PTB, a tree-in-bud pattern had high sensitivity (71.0%); and small centrilobular pulmonary nodules, a tree-in-bud pattern, consolidation, and especially cavitation all had high specificity (80.8%, 73.1%, 73.1%, and 84.6%, respectively), with PPVs of 79.2%, 75.9%, 73.1%, and 77.8%, respectively.

We found that MDCT presented reliable predictive measures for the main tomography findings in the diagnosis of PTB, therefore being a safe tool for diagnosing the disease in patients with clinical suspicion of the disease, especially when a specific set of characteristic tomography findings (small pulmonary nodules, a tree-in-bud pattern, consolidation, and cavitation) is found in patients residing in an area of high PTB prevalence. The use of MDCT can enable prompt initiation of drug treatment, reducing the mortality associated with the disease and minimizing its spread within the community.

Our study has certain limitations, including the small sample size, which can limit the generalizability of the results. In addition, the retrospective nature of the study limited its ability to identify temporal changes. Furthermore, all MDCT scans were evaluated by the same observer and that singular perspective could have influenced the conclusions. These limitations should be considered when interpreting the results, underscoring the need for future research to address these issues and provide a more comprehensive view of the topic.

## CONCLUSION

MDCT proved useful in the diagnosis of PTB. It also appears to be a suitable tool for selecting candidates for more complex, invasive examinations from among smear-negative patients in whom there is a high suspicion of PTB.
